# Who is accessing community lateral flow device testing and why? Characteristics and motivations of individuals participating in COVID-19 community testing in two English local authority areas

**DOI:** 10.1186/s12889-022-12986-4

**Published:** 2022-03-25

**Authors:** Michael N. Dalili, Jo Long, Emma Wadley, Jack Sloan, Andrew Cross, Kyla H. Thomas, Gemma Morgan

**Affiliations:** 1 Bristol Medical School, Population Health Sciences, University of Bristol, Bristol, UK; 2 South Gloucestershire Council, Yate, UK; 3 North Somerset Council, Clevedon, UK

**Keywords:** COVID-19, Coronavirus, SARS-CoV-2 virus, Pandemic, Lateral flow device, Lateral flow testing, Rapid testing, England, Local authority, Community testing

## Abstract

**Background:**

Antigen testing using lateral flow devices (LFDs) plays an important role in the management of the novel coronavirus pandemic of 2019 (COVID-19) by rapidly identifying individuals who are asymptomatically carrying high levels of the virus. By January 2021, LFD community testing sites were set up across English local authority areas to support the management and containment of regional COVID-19 cases, initially targeting essential workers unable to work from home during the national lockdown. This study aimed to examine the characteristics and motivations of individuals accessing community LFD testing across two local authority areas (LAAs) in the South West of England.

**Methods:**

Data were collected as part of a service evaluation from December 22^nd^ 2020 until March 15^th^ 2021 for two LAAs. Demographic and postcode data were collected from an online test appointment booking platform and the National Health Service testing service online system, with data accessed from Public Health England. An online survey was sent to individuals who made a testing appointment at an LAA1 site using the online booking platform, consisting of 12 questions to collect data on individual’s motivations for and experiences of testing.

**Results:**

Data were available for individuals who completed 12,516 tests in LAA1 and 12,327 tests in LAA2. Most individuals who engaged with testing were female, working age, white, and worked as early years or education staff, health and social care staff, and supermarket or food production staff. 1249 individuals completed the survey with 60% of respondents reported getting tested for work-related reasons. Individuals first heard about LFD testing through various channels including work, media, and word of mouth, and decided to get tested based on the ease and convenience of testing, workplace communications, and to identify asymptomatic cases to help stop the spread. Most tests were completed by individuals living in less deprived areas based on national deciles of deprivation.

**Conclusions:**

While national and local COVID-19 testing strategies have evolved, community and personal LFD testing remains a crucial pillar of the testing strategy. Future studies should collect quantitative and qualitative data from residents to most effectively shape testing offers based on the needs and preferences of their population.

**Supplementary Information:**

The online version contains supplementary material available at 10.1186/s12889-022-12986-4.

## Background

Rapid diagnostic tests, such as antigen testing using lateral flow devices (LFDs), have contributed to the management of the Severe Acute Respiratory Syndrome Coronavirus-2 (SARS-CoV-2) novel coronavirus pandemic of 2019 (COVID-19). While laboratory-based polymerase chain reaction (PCR) tests are considered the gold-standard for diagnosing clinical COVID-19 infection [1, 2], they are costly and time consuming. LFDs are cheap, portable, fairly easy to administer or self-administer, and can deliver results in 15–30 min [3]. They can rapidly identify those individuals who are asymptomatically carrying high levels of the virus so that they may self-isolate, breaking chains of transmission [3]. Previous studies have suggested that LFDs could be sufficiently sensitive to detect large numbers of positive cases quickly, including the majority of cases that led to onward transmission [4–6].

However, questions have been raised regarding the reliability of these tests and whether they are appropriate to use as part of mass community testing programmes [7]. Concerns have been raised about the varying levels of false negatives [8–11], a higher absolute number of false positives when community prevalence of SARS-CoV-2 is low [4], and the lack of clarity in public messaging around the interpretation of test results [12, 13]. Other studies stress the need for LFD testing to be used by individuals very regularly in order for them to be an effective screening tool, as their accuracy is affected by viral load [6, 14]. Despite this, LFDs have been a key part of the large-scale community testing carried out as part of the UK government’s COVID-19 2020/2021 Winter plan to manage virus transmission [15].

The LFD rapid testing programme evolved amidst shifts in national and local strategies and policy. During the initial phase of the LFD rapid testing programme from November 2020 to early December 2020, many local authorities were under ‘Tier restrictions’ that allowed early years, schools and universities, retail, gyms, personal care, and outdoor sports to remain open. At this time, secondary school children and essential workers were considered priority groups for community testing based on elevated infection rates among these groups. Therefore, initially the focus for local authority community testing was on essential workers and controlling place-based outbreaks. On 4^th^ January 2021, a national lockdown was announced in response to an increase in COVID-19 infections and hospitalisations. Schools were closed and a “stay at home” (including work from home) policy was implemented. The target groups for community testing were reassessed as it became clear that some groups were now at much lower risk due to the directive and the closure of retail, leisure, and school sites. By the end of January 2021 LFD community testing sites were set up within high infection rate communities from across local authorities to support the management and containment of COVID-19 cases in the regions, initially targeting essential workers unable to work from home during the national lockdown. This study aimed to examine the characteristics and motivations of individuals accessing community LFD testing across two local authority areas (LAAs) in the South West of England.

## Methods

### Data collection

Data were collected as part of a service evaluation of the LFD community testing provision conducted across two LAAs in the South West of England. Residents from and individuals working in these two LAAs were offered community LFD testing. Delivery of the LFD testing site programme for both local authorities (LAs) was contracted to an external event management company. Testing locations included large sports halls based in leisure centres and large local community halls that were accessible and included parking. Upon arriving at a testing site, individuals registered online with the National Health Service (NHS) testing service and provided a contact email and/or a telephone number that was used to deliver the individual’s result via the central system. Innova LFDs were the tests used at these testing sites. These devices have been found to have a sensitivity of 80% or higher for individuals with high viral loads, even among inexperienced users [16]. Their specificity is also high, above 99%, including for novice self-testers.

### Quantitative data

Occupation data were collected from the online booking platform (simplybook.cc) that individuals used to book testing appointments, managed by the external event management company. Appointments that were booked but later cancelled were excluded. For individuals who went on to get tested, further demographic data and postcode information were collected from the NHS testing service online system and accessed by LAs via Public Health England’s Power BI portal (test line list data from “Line List Positive Tests” and “Line List Negative and Void Tests”). We also used test line list data to investigate testing uptake by 2019 national Index of Multiple Deprivation (IMD) deciles, assigned based on 2011 lower super output areas (LSOA) for both LAAs. Test line list data also include data for individuals who were tested multiple times as we were unable to deduplicate this data. Data presented here were reported from LAA1 from January 18^th^ 2021 up to March 14^th^ 2021 (March 15^th^ for occupation data) and from LAA2 from December 22^nd^ 2020 up to February 26^th^ 2021.

### Qualitative data

A survey was sent via email to individuals who made a testing appointment at an LAA1 site using the online booking platform. The survey was hosted on SnapSurveys (https://www.snapsurveys.com/) and was sent directly to 5215 unique email addresses on March 8^th^ 2021, with a reminder sent on March 12^th^ before the survey closed on March 14^th^ after seven days. The survey was developed using SnapSurveys software (Snap 11) and was uploaded and hosted by SnapSurveys online. Survey data were collected and automatically downloaded to LAA1 filestores. The survey consisted of 12 questions to collect data on individual’s motivations for and experiences of testing (see Appendix 1). Only individuals who made an online booking appointment were contactable for this purpose, and they received a single survey request irrespective of the number of bookings they had made. Survey completion was optional, and respondents were informed that all personal details would be removed and their responses would be kept anonymous. Survey response data were analysed using thematic analysis, with emergent themes represented as response categories. Responses were assigned to themes based on the central point of their content.

## Results

### Demographic characteristics

Demographic characteristics available for individuals who completed 12,516 LFD tests in LAA1 and 12,327 LFD tests in LAA2 are presented in Table 1. There was high female engagement with LFD testing across LAAs. Proportions of tested individuals by ethnicity were similar to 2011 census estimates.

**Table 1:** Demographic characteristics of individuals who participated in LFD testing by local authority (LAA)

	**Female n (%)**	**Age n tests (%)**									**Ethnicity n (%)**		
		10–19	20–29	30–39	40–49	50–59	60–69	70–79	80–89	90–99	White	Ethnic Minority	No information
**LAA1**	7701 (62)	520 (4.2)	2977 (23.8)	2870 (23.0)	2596 (20.8)	2327 (18.6)	980 (7.8)	180 (1.4)	42 (0.3)	1 (0.008)	11,696 (93.5)	597 (4.8)	223 (1.8)
**LAA2**	7355 (60)	1297 (10.5)	2202 (17.9)	2242 (18.2)	2521 (20.5)	2714 (22.0)	985 (8.0)	270 (2.2)	53 (0.4)	6 (0.1)	11,677 (94.7)	234 (1.9)	416 (3.4)

**Fig 1:**
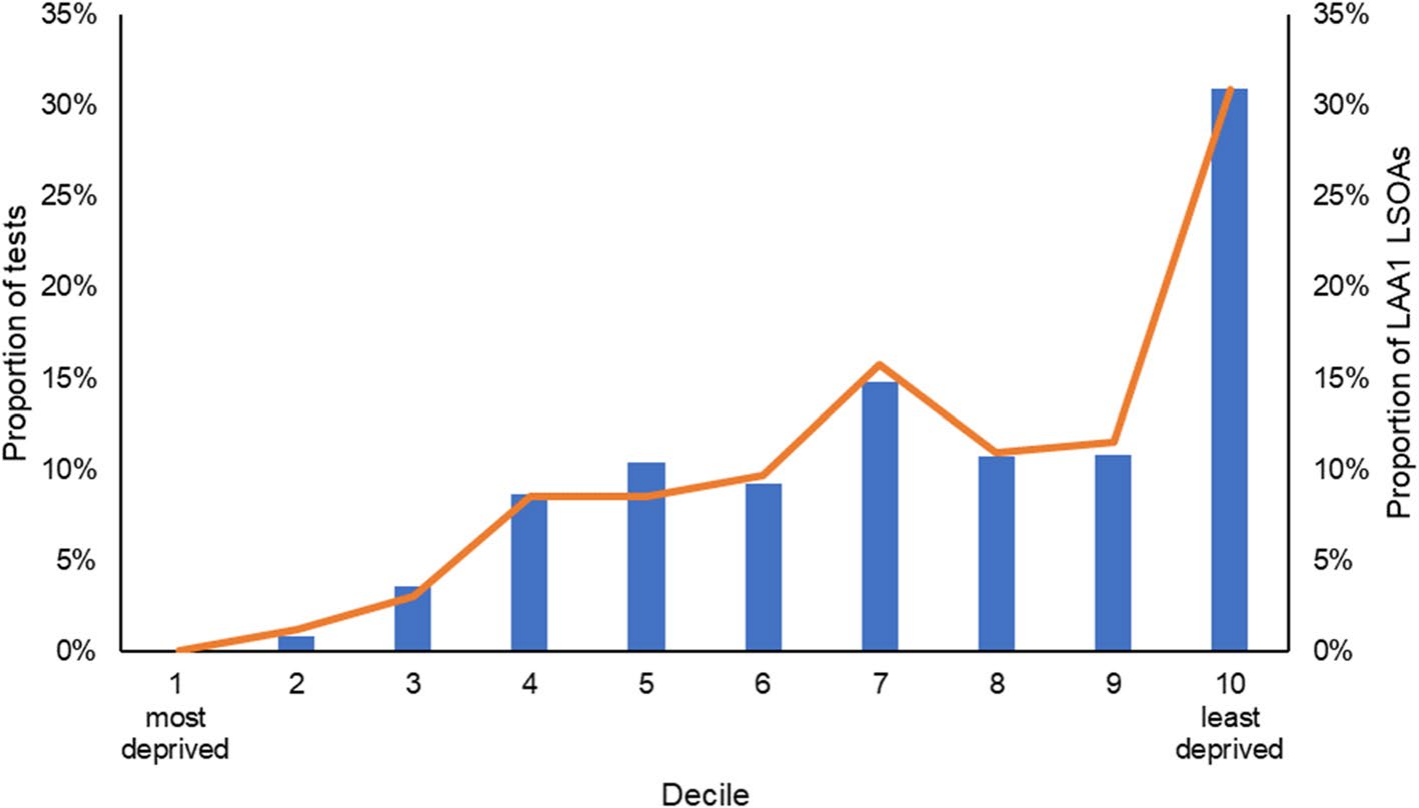
Proportion of tests conducted at LAA1 testing sites and proportion of LAA1 LSOAs by National Index of Multiple Deprivation Decile

Based on national IMD deciles, the highest proportion of LAA1 tests (31%) were attributed to individuals from LAA1 LSOAs in the least deprived decile (see Fig. 1). The majority (77%) of tests were taken by residents from LSOAs among the less deprived (≥ 6^th^) national deciles, with no tests in the most deprived national decile (note that LAA1 has no LSOAs which fit into the most deprived national decile).

**Fig 2:**
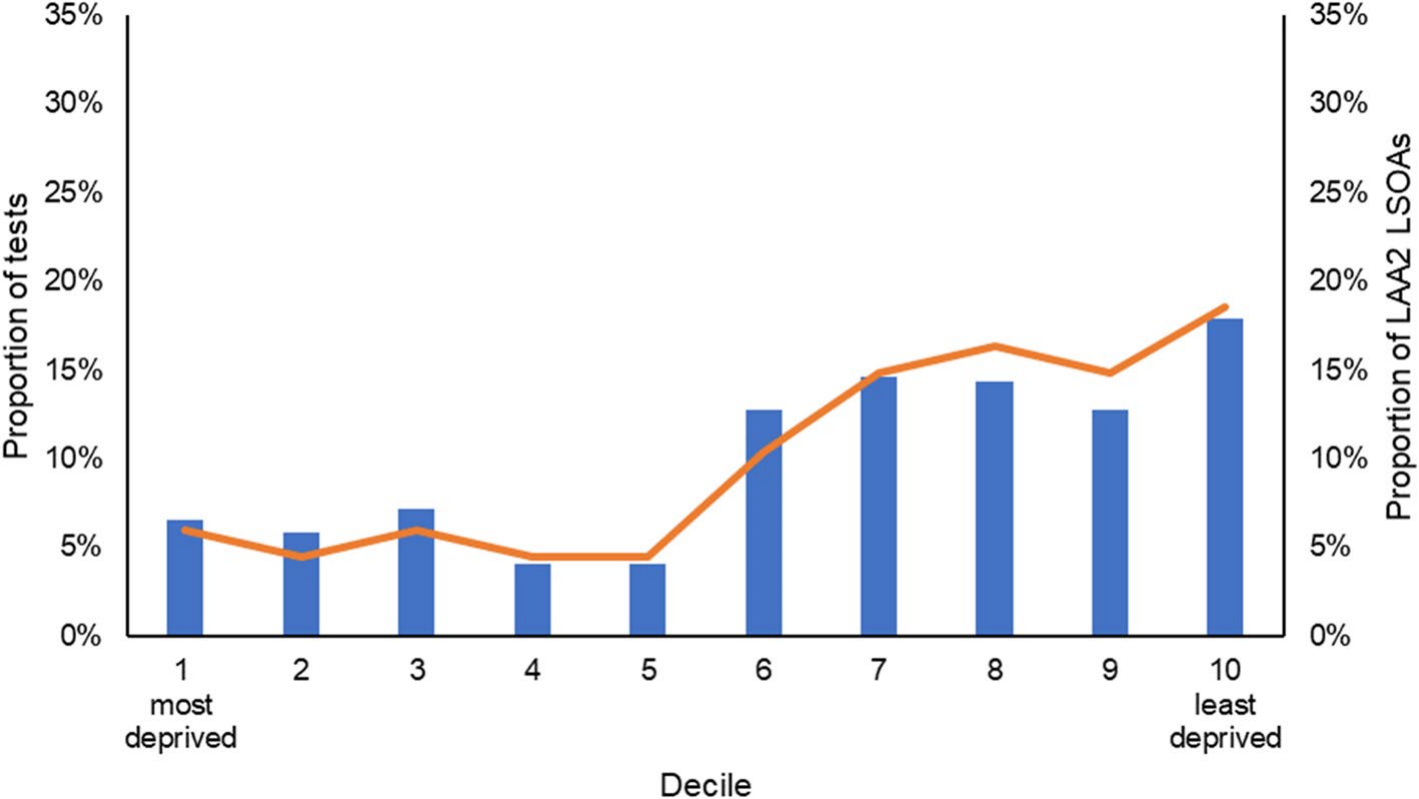
Proportion of tests conducted at LAA2 testing sites and proportion of LAA2 LSOAs by National Index of Multiple Deprivation Decile

Similarly, the highest proportion of LAA2 tests (18%) were also attributed to individuals from LAA2 LSOAs in the least deprived decile (see Fig. 2). Additionally, like LAA1, most tests (72%) were taken by residents from LSOAs among the less deprived (≥ 6th) national deciles.

### Occupations

Occupation data from individuals who booked 16,010 test appointments in LAA1 and 15,515 test appointments in LAA2 are presented in Table 2. Among reported occupations, early years or education staff and health and social care staff represented the occupations that made the most repeat bookings in LAA1 while early years or education staff and supermarket or food production staff made the most repeat bookings in LAA2. While reporting occupation when booking an appointment was mandatory for LAA1, it was not for LAA2, accounting for the large proportion of “I do not wish to say” responses.

**Table 2:** Reported occupations of individuals who booked tests by local authority (LAA)

Occupation	LAA1 Frequency (%)	LAA2 Frequency (%)
Early years or education staff	2676 (19.6)	716 (9.2)
Emergency services staff	874 (6.4)	492 (6.4)
Funeral and crematorium staff	26 (0.2)	9 (0.1)
Health and social care staff including personal carers, social workers, and health visitors	1905 (13.9)	390 (5.0)
I do not wish to say	2168 (15.9)	3356 (43.3)
Key public services such as justice system, faith work, journalism	257 (1.9)	203 (2.6)
Library staff	49 (0.4)	12 (0.2)
Other customer-facing council staff unable to work from home	308 (2.3)	103 (1.3)
Other essential work not listed above	3731 (27.3)	2090 (27.0)
Supermarket or food production staff	836 (6.1)	630 (8.13)
Transport worker including parking management	572 (4.2)	295 (3.8)
Voluntary sector staff in public-facing roles	170 (1.2)	136 (1.8)
Waste management staff	101 (0.7)	68 (0.9)

Survey respondents (*n* = 1234) reported other categories of occupations not captured by the options on the online booking platform, including banking (2%), postal workers (1%), police (6%), and building and construction workers (2%), as well as those working in other people’s homes such as estate/lettings agents (1%), utility and broadband service engineers (1.5%), and domestic cleaners (1%).

### Motivations and communications

1249 individuals who booked a testing appointment at a LAA1 site completed the survey for a response rate of 24%. Most respondents (60%, *n* = 748) reported they were getting tested for several work-related reasons, including because it was a requirement for their job (14%), they had been ‘advised’ or ‘encouraged’ by their managers to get tested (4%), or that they were getting tested because they worked in the community or with vulnerable people (14%).

**Fig 3:**
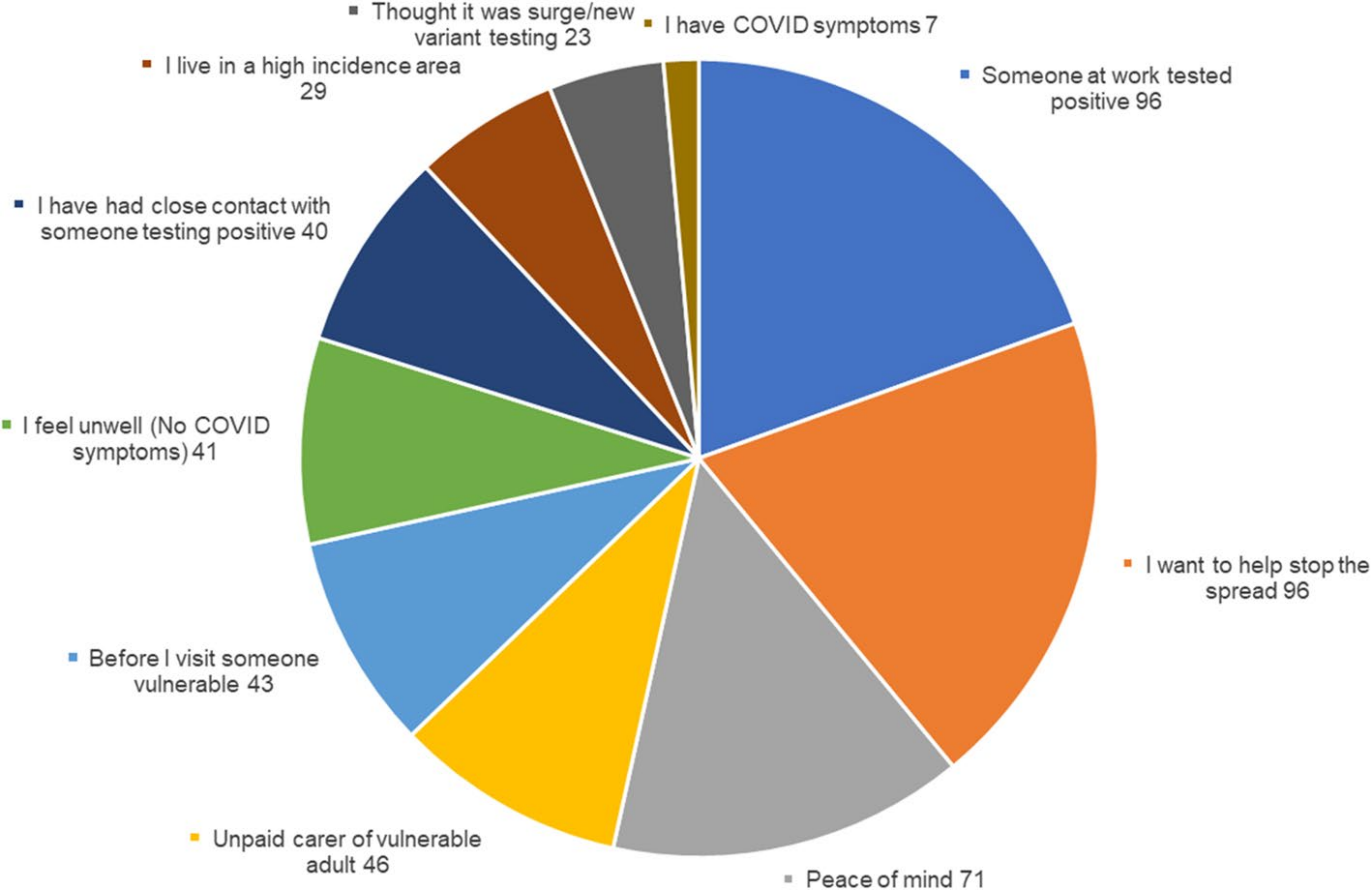
Number of responses for non-work reasons for getting tested

Reasons given by the remaining 40% of respondents can be seen in Fig. 3. Some respondents visited the LFD testing sites because they felt unwell with COVID-19 symptoms, despite the booking platform requiring individuals to confirm they did not have symptoms in order to book a test. Survey results also indicated that a small number of those booking LFD tests thought they were booking tests as part of the rapid asymptomatic surge testing programme deployed in LAA1 in response to a new COVID-19 ‘variant of concern’.

**Fig 4:**
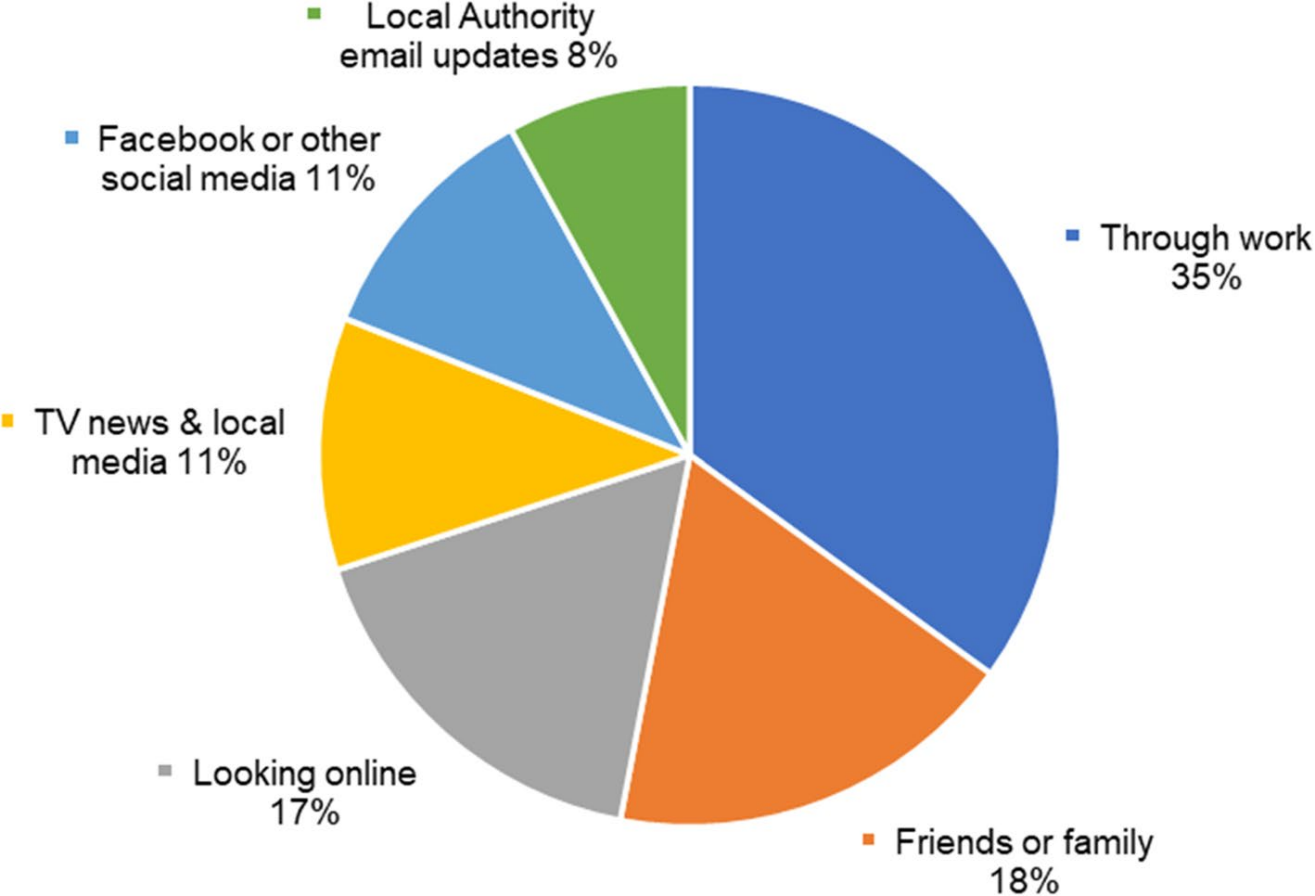
Where individuals first heard they could get a test in their area

The survey also asked where individuals had first heard that they could get a rapid test in their local area (see Fig. 4). Responses (*n* = 1244) were spread quite evenly across various platforms of communication. Some responses (excluded due to small numbers) included those that referred to individuals such as local MPs, named police officers, or GPs, and from calling 111 (NHS medical help number) or 119 (COVID-19 testing service number). A small number of survey respondents also stated that testing was ‘not well known about’ and ‘poorly advertised’, and 35 respondents reported they thought it should be more widely publicised.

**Fig 5:**
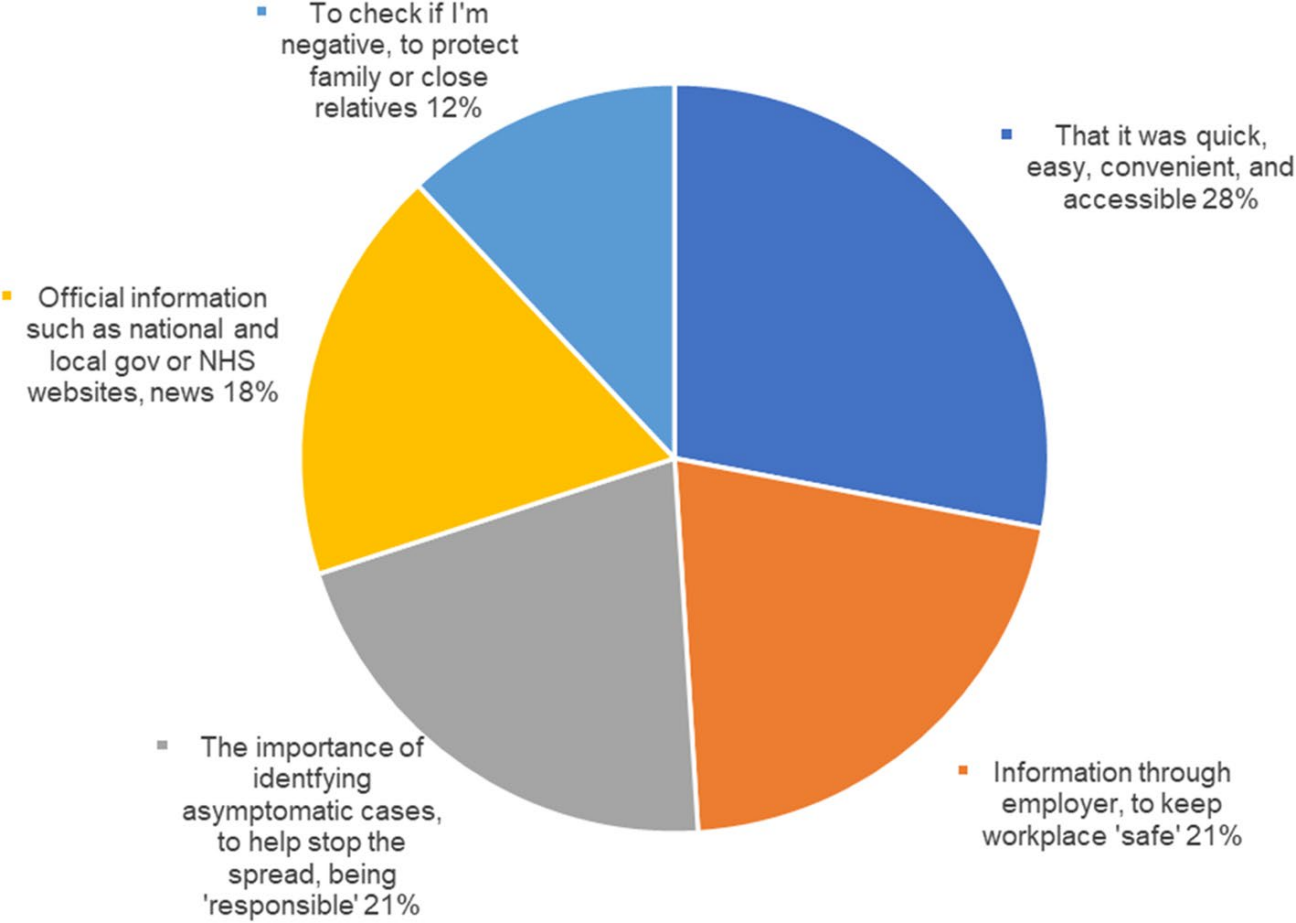
Information that helped individuals decide to get tested

The survey also asked ‘What information helped you to decide to get tested?’, with the most common response (28% of respondents) being the speed, ease, and convenience of testing (see Fig. 5).

## Discussion

This descriptive study aimed to examine the characteristics and motivations of individuals accessing community LFD testing across two LAAs in the South West of England. We found most individuals who engaged with testing were female, between the ages of 20 and 59 (working age adults), white, and worked as early years or education staff, health and social care staff, and supermarket or food production staff. Most individuals got tested for work-related reasons, as well as having been in contact with infected individuals, feeling unwell or symptomatic, or to stop the spread of the virus. Individuals first heard about LFD testing through various channels including work, media, and word of mouth, and decided to get tested based on the ease and convenience of testing, workplace communications, and to identify asymptomatic cases to help stop the spread.

Increased female engagement with LFD testing across LAAs was likely due to the gender disparity in some of the ‘essential worker’ occupation categories with known limited access to testing at work. According to 2020 ONS data [17], women make up 83% of ‘care workers and home carers’, and 98% of ‘nursery workers and childminders’ in the UK. While the proportions of ethnic minorities among individuals accessing community LFD testing were similar to those of the LAA populations’ 2011 census ethnic distribution, more recent data from 2016 [18] show an increase in ethnic minority residents (by 1.5% in LAA1, and by 0.3% in LAA2). Therefore, our data suggest an underrepresentation of ethnic minorities accessing testing for these LAAs. While the greatest proportion of tests was taken by individuals living in LSOAs among the least deprived national IMD decile for both LAAs, tests were proportionally distributed relative to how many of each LAA’s LSOAs are in each national decile. Deciles with higher proportions of tests reflect a larger number of LAA LSOAs that fell into that IMD decile. For example, 31% of LAA1’s LSOAs (*n* = 51) were in the least deprived (10^th^) national decile, where 31% of all LAA1 tests were conducted. Our occupation data suggest communications targeting essential workers to get tested were successful, as individuals in these roles represented large proportions of our sample. However, our survey results indicated that our occupation response categories were lacking, as several occupations were identified that we had been unable to capture such as police, construction workers, and cleaners. There were also communication challenges during the study period. Changes due to shifts in national and local policy and strategies resulted in inconsistent messaging with regards to priority groups for, and frequency of, community testing. For example, in LAA1, some communications shared with community groups at the start of the testing offer presented confusing information. One presentation initially stated “anyone can access these tests” but went on to specify that “we are asking those who cannot work from home and who deliver key services to the community specifically to use this opportunity” to get tested. Communications to another community group suggested the prioritisation of testing “with critical workers and volunteers in roles which bring them into contact with the community being prioritised with the offer of weekly slots” but also stated that local residents were encouraged to take up the testing offer and stressed the importance of asymptomatic testing. An LAA1 internal staff news item from January 27^th^ 2021—February 2^nd^ 2021 provided clearer messaging, stating they were “targeting those who can't work from home in the current lockdown and those in areas with higher rates of Covid” for testing, and a further news item “Do I need to get Covid tested?” that ran from February 10^th^-15^th^ that stated “The Lateral Flow Test is intended to be completed regularly by those living in areas with high infection rates and critical workers in the community”. However, when LAA1 opened their third testing site on February 22^nd^ 2021, internal communications stated testing was recommended for “staff who cannot work from home and who come into contact with colleagues and the public in order to do their jobs” and made no mention of getting tested based on infection rates in local communities. Promotion of LFD testing was also problematic when surge testing programmes were deployed in LAA1. Due to concerns that the different types of tests could potentially confuse residents, surge testing messaging was prioritised by the LAA1 communications team during those periods.

Our findings regarding who is accessing testing are similar to those from a recent study reporting findings on social and spatial inequalities in uptake and case-detection of a community LFD testing pilot in Liverpool for asymptomatic residents that ran between 6^th^ November 2020 to 31^st^ January 2021 [18]. The authors also reported higher uptake among women and lower uptake among ethnic minority groups. However, while they found lower uptake and more positive tests among those living in the most deprived areas, we did not. However, we were unable to investigate this with the same spatial sensitivity and precision, instead relying on exploring number of tests by postcode data self-reported during test registration. Similar to our findings, a rapid scoping review that thematically analysed the findings of 47 studies to investigate motivations and barriers to seeking, accessing, and undertaking testing found that perceived convenience of testing site and endorsement from employers, educational institutions, peers, and/or colleagues encouraged the uptake of testing [19]. They also found that the perceived benefits of testing included to protect family, colleagues, and others in the community by reducing the spread of COVID-19, information about their disease status, and to contribute to scientific research and public management of the pandemic. In a recent study, researchers conducted interviews and focus groups with 223 staff, students, pupils and household members from schools, a university, and a community healthcare NHS trust to explore the experiences of individuals who took part in a weekly COVID-19 pilot testing programme [20]. Like our study, they found that communication, a sense of community, and convenience were crucial to people’s engagement with the testing programme, with participants feeling reassured by and proud of their participation in the programme to help manage the pandemic.

There were some limitations of our study. Total sample sizes differed by outcome as some individuals were tested without booking an appointment, some data for individuals who lived outside the LAAs were unavailable, and there was a delay in receiving test line data relative to booking platform data. We were only able to survey residents of LAA1, and despite many residents completing the survey, the survey’s low response rate is a limitation that should be considered when interpreting its findings. Additionally, our survey was not designed using any specific theoretical model, which could be considered for future studies (e.g., health belief model). Finally, due to the evolving and serious nature of the pandemic, services such as community testing have been introduced and initiated at pace. Consequently, evaluation has not always been built in from the outset, rendering it difficult to comprehensively assess the potential impact of these services and their ability to reach the most deprived and at-risk individuals within our populations. Future services should develop robust evaluation plans prior to launching, and ensure monitoring occurs throughout service delivery so that the service can change and adapt at pace when necessary to meet the needs of its target population. Additionally, while the Innova LFD tests used in this study were shown to have high sensitivity and specificity [16], their sensitivity drops for individuals with lower viral loads. Given the absolute number of false positives will be high when community prevalence of SARS-CoV-2 is low, their use should be continually evaluated, particularly for mass testing [4].

## Conclusions

This is one of the first studies to investigate the motivations of individuals accessing community LFD testing, which we believe provides valuable insight that can be used to shape communication and deployment strategies to encourage testing uptake. While national and local COVID-19 testing strategies have evolved since the study period, LFD testing remains a crucial pillar of the testing strategy. Future COVID-19 testing studies should continue to collect rich quantitative and qualitative data from residents to most effectively shape national and local testing offers to adapt to the needs and preferences of the target population. Studies should also consider surveying individuals who were not interested in or refused COVID-19 testing to better understand how to promote and increase uptake of the testing offer for the entire population.

## Supplementary Information

**Additional file 1.**  Online survey questions sent to individuals who made a COVID-19 community LFD testing appointment at an LAA1 site.

## Data Availability

The datasets used and/or analysed during the current study are available from the corresponding author on reasonable request.
