# Supplementary material for: Who is accessing community lateral flow device testing and why? Characteristics and motivations of individuals participating in COVID-19 community testing in two English local authority areas
Source: BMC Public Health. 2022 Mar 25;22:588. doi: 10.1186/s12889-022-12986-4 (PMC8956328; doi:10.1186/s12889-022-12986-4)
Supplement: Supplementary file 1 — Additional file 1. Online survey questions sent to individuals who made a COVID-19 community LFD testing appointment at an LAA1 site. [file 12889_2022_12986_MOESM1_ESM.docx]

# Appendix 1 - Survey

This survey is **only** for people who booked a Covid-19 rapid test (lateral flow test) at **[LAA1 sites]**

There are 12 questions which should take you no more than two minutes to complete. Please fill in your answers with as much detail as you can. Please do not include personal details such as names or addresses. All personal details will be removed to protect your privacy, and all responses will be kept anonymous.

1. How did you first hear about rapid tests for Covid-19 in [LAA1]? TEXT BOX
2. Did you book a test for just yourself or for other people? - please state their relationship to you (no names please) TEXT BOX
3. Why did you book a Covid-19 rapid test? TEXT BOX
4. What information helped you to decide to have a Covid-19 rapid test? TEXT BOX
5. Did your Covid-19 rapid test result affect what you did afterwards? (whether negative, positive or void) YES/NO

Can you explain why? TEXT BOX

1. What is your occupation? TEXT BOX
2. What test site did you use? [LAA1 sites]
3. How easy or difficult was it to book a Covid-19 rapid test online – VERY EASY / QUITE EASY / NEITHER EASY OR DIFFICULT / QUITE DIFFICULT/ VERY DIFFICULT

Can you explain why? TEXT BOX

1. What was your overall experience at the testing site? – EXCELLENT / GOOD / NEITHER GOOD OR BAD, / POOR ?

Can you explain why? TEXT BOX

1. Do you plan to, or are you already doing regular Covid-19 rapid testing? YES / NO
2. If yes how frequently will you be booking a Covid-19 rapid test? MORE THAN TWICE WEEKLY / TWICE WEEKLY / WEEKLY / LESS THAT WEEKLY
3. Do you have any other comments or thoughts on your experience of rapid testing? TEXT BOX
